# Clinical Factors Affecting the Quality of Life of Women With Endometriosis

**DOI:** 10.1111/jan.16607

**Published:** 2024-11-11

**Authors:** Agnieszka Bień, Aleksandra Pokropska, Joanna Grzesik‐Gąsior, Magdalena Korżyńska‐Piętas, Marta Zarajczyk, Ewa Rzońca, Krzysztof Jurek

**Affiliations:** ^1^ Chair of Obstetrics Development, Faculty of Health Sciences Medical University of Lublin Lublin Poland; ^2^ Center of Gynecology and Obstetrics GEMELLI Private Health Care Cracow Poland; ^3^ State University of Applied Sciences in Krosno Krosno Poland; ^4^ Department of Obstetrics and Gynecology Didactics, Faculty of Health Sciences Medical University of Warsaw Warsaw Poland; ^5^ Institute of Sociological Sciences John Paul II Catholic University of Lublin Lublin Poland

**Keywords:** clinical factors, endometriosis, midwifery, nursing, obstetrics and gynaecology, quality of life, reproductive health, women's health

## Abstract

**Aim:**

The study aimed to analyse selected clinical data affecting the quality of life of women with endometriosis.

**Design:**

A cross‐sectional study.

**Method:**

The study was conducted in 2020–2022 among 425 women with endometriosis receiving health care. A diagnostic survey method with a questionnaire technique was used. The research tools are the Endometriosis Health Profile and a standardised interview questionnaire.

**Results:**

Infertility, medical treatment and sexual intercourse were the dimensions of quality of life most poorly rated by respondents with endometriosis. Lower quality of life was linked to the presence of painful periods, the presence of pain during intercourse and having surgery for endometriosis.

**Conclusion:**

The main clinical variables influencing the quality of life of women with endometriosis include problems with getting pregnant, the necessity for long‐term therapy and sexual problems caused by the condition. The occurrence of painful menstruation, dyspareunia and having undergone surgery for endometriosis is associated with lower ratings of women's general quality of life.

**Implications for the Profession:**

The study will help to develop a more holistic approach to caring for endometriosis patients. This will result in a better diagnosis, individualised therapy and psychological support, all of which will enhance the overall quality of life. The study's results may impact the development of health policies, endometriosis support services for women and public awareness initiatives.

**Impact:**

The study highlighted key determinants affecting health‐related quality of life for women with endometriosis. The analysis of clinical data revealed that infertility, treatment and dyspareunia are the dimensions of quality of life most poorly rated by women with endometriosis. These findings are significant for those providing care to women with endometriosis, as well as for policymakers responsible for the organisation of health care systems.

**Reporting Method:**

The STROBE guidelines.

**Patient or Public Contribution:**

No patient or public contribution.

**Summary**

- What is known about this topic?
  ○ Endometriosis is a chronic, hormone‐dependent disease in which endometrial tissue develops outside the uterus, leading to chronic inflammation, pain and infertility.
  ○ Diagnosing endometriosis can be a lengthy and complex process, as the symptoms can vary in severity and may not always be immediately recognisable.
  ○ Endometriosis negatively affects the quality of life, well‐being, family functioning and workplace environment for women. Those experiencing this condition may have complex needs and require long‐term support.
- What does this paper add?
  ○ The main clinical factors affecting the quality of life of women with endometriosis include fertility issues, the necessity for long‐term treatment and challenges in sexual life.
  ○ Heavy menstrual bleeding is one of the most significant problems for women with endometriosis.
  ○ The occurrence of painful menstruation, dyspareunia, and undergoing surgery due to endometriosis is associated with lower quality of life ratings among affected women.
- Implications for policy and practice
  ○ Improving the quality of life for women with endometriosis requires better access to effective treatments, including hormonal and pain‐relief medications, as well as alternative therapies.
  ○ Increasing the availability of therapeutic sessions and support groups for women with endometriosis is crucial to help them cope with the emotional and psychological impacts of the disease.
  ○ Awareness campaigns about endometriosis could enhance understanding of the condition, its symptoms, and its effects on women's daily lives and work. It would also be beneficial to consider policies that facilitate medical leave for women experiencing severe menstrual pain and/or heavy bleeding, as well as shorter working hours or the possibility of remote work.

## 1 Introduction

Endometriosis is a hormone‐dependent disease of a chronic nature that usually involves the pelvic organs such as ovaries, fallopian tubes ligaments and peritoneal surfaces. It is less common in other parts of the body, such as the gastrointestinal tract, urinary tract, lungs, central nervous system and other organs. This is a disease characterised by endometrium‐like epithelium and/or lining located outside the uterine cavity and mucosa, which undergoes a cyclical proliferation and breakdown similar to the endometrium. An inflammatory process usually accompanies the disease (Klemmt and Starzinski‐Powitz 2018; Zondervan, Becker, and Missmer 2020). The first symptoms of endometriosis can appear before the age of 20 and among them are mainly chronic pelvic pain, painful menstruation, dyspareunia, painful micturition and defecation (Taylor, Kotlyar, and Flores 2021; Sachedina and Todd 2020). Although it is possible to screen women at high risk for endometriosis, it often takes as long as 8–10 years to make a diagnosis. The delay may be caused by non‐obvious symptoms of variable severity, which can influence misdiagnosis. Endometriosis symptoms sometimes are similar to menstrual cycle complaints, which can cause them to be ignored. Some women may avoid complaining about their symptoms, which can delay a consultation with gynaecologists who specialise in endometriosis treatment (Zondervan, Becker, and Missmer 2020).

Endometriosis affects approximately 10% of women of reproductive age. However, in some women, the disease has a subclinical course, making it difficult to determine the exact prevalence in the general population. Endometriosis can be understood as a condition with a variable course and effects at different stages of life. There is often a long delay in the diagnosis of the disease after the onset of symptoms, as well as the persistence and recurrence of symptoms despite treatment (Vermeulen et al. 2021). Endometriosis can significantly affect fertility by causing adhesions or fibrosis of the fallopian tubes, inflammation of the pelvic tissues and changes in the uterine environment. Up to 50% of women with infertility problems have been diagnosed with foci of endometriosis (Vermeulen et al. 2021; Horton et al. 2019; Hodgson et al. 2020).

## 2 Background

The diagnosis of endometriosis is based on clinical examination, diagnostic imaging and surgery such as laparoscopy (Koninckx et al. 2021; Horne and Missmer 2022; Rolla 2019). The American Society for Reproductive Medicine (rASRM) has developed a four‐step scale to assess the severity of endometriosis. The scale is based on the number, type and size of endometriosis foci, as well as the degree of infiltration of surrounding tissues and the presence of additional lesions like adhesions or tumours, the severity of the disease is not related to the pain intensity. The ASRM scale is used to diagnose the severity of endometriosis during surgery (Vermeulen et al. 2021).

In addition to the patient's history, gynaecological examination and diagnostic imaging, laboratory tests can help diagnose the disease. One non‐invasive test is the determination of CA‐125 and BDNF (brain‐derived neurotrophic factor) levels in the blood. While the CA‐125 marker does not confirm the presence of endometriosis, elevated levels can suggest advanced stages III and IV. In combination with BDNF determination, it helps identify Stages I and II of the disease (Jafarabady et al. 2024; Herranz‐Blanco et al. 2023). Another test is an endometrial biopsy‐based test that identifies BCL‐6 (B‐cell lymphoma 6), a protein biomarker associated with inflammation. A positive test indicates high levels of BCL‐6, which may suggest endometriosis but does not conclusively confirm it (Nezhat et al. 2022). The EndoRNA qRT‐PCR test, a minimally invasive endometriosis test, has been commercially available in Poland since 2023. The test involves taking a swab from the uterine cavity and determining the expression level of the gene encoding the protein fucosyltransferase 4 FUT4, which is significantly higher in patients with endometriosis. This test is used to confirm or exclude the disease (Żeberkiewicz et al. 2022).

Endometriosis is one of the most common gynaecological conditions, affecting a significant number of women. Considering the scale of the problem, it is worth implementing actions related to its prompt diagnosis and effective treatment. The symptoms of endometriosis have a significant impact on women's professional and social lives and generate high costs related to treatment and loss of productivity. Gaining better knowledge of the clinical factors affecting quality of life could lead to more effective management of the disease and lower costs for health systems worldwide. International studies, a combination of insights from patients as well as professionals from different countries, enable the exchange of experiences and incorporate a wide range of perspectives on the care of patients with endometriosis. Such activities help to create integrated therapeutic approaches and support tailored to individual women's needs. This diversity not only improves the quality of recommendations but also fosters greater acceptance in different health care systems around the world (Schleedoorn et al. 2016; Hirsch et al. 2018; Taylor, Kotlyar, and Flores 2021).

Endometriosis is treated symptomatically, due to the lack of knowledge of the pathogenesis of the disease. The proposed treatment: analgesic, hormonal and surgical should be adapted to the woman's condition, the intensity of symptoms, daily functioning, the patient's preferences and plans to become pregnant (National Guideline Alliance (UK) 2017). Beyond the physical symptoms, endometriosis can have a significant impact on women's overall quality of life, including their mental health, relationships and career. Women may have complex needs and require long‐term support (Bień et al. 2020; Missmer et al. 2021; Pontoppidan, Olovsson, and Grundström 2023). Constant discomfort and pain often lead to physical limitations, affecting daily activities, work and relationships. Endometriosis can strain relationships with partners and the pain associated with sexual intercourse may reduce sexual desire (Melis et al. 2015). Struggling with a chronic disease, especially one that can affect fertility, can lead to frustration, anxiety and depression. Symptoms of endometriosis and ailments associated with it can cause social isolation, which affects women's mental health negatively (Arslan, Kocaöz, and Kızılırmak 2024; Zarbo et al. 2022; Vitale et al. 2017).

## 3 The Study

### 3.1 Aim

The study aimed to analyse selected clinical data related to the diagnosis of the disease affecting the quality of life of women with endometriosis. The selection of clinical data was dictated by their relevance to the diagnosis, treatment and impact of endometriosis on women's daily functioning and quality of life. The analysis will allow a better understanding of the problem and the identification of areas that need special attention in clinical practice.

## 4 Methods

### 4.1 Design

The study was conducted between 2020 and 2022 in health clinics in the Lublin Province (Poland). Questionnaires were distributed among all women with a confirmed diagnosis of endometriosis, who came to the clinic because of complaints related to the disease or for follow‐up appointments, such as for a PAP smear. In total, 425 women with endometriosis took part in the project. All patients were informed that their participation was voluntary and anonymous, that they could withdraw from the study at any moment and that all data would be used for research purposes only.

### 4.2 Inclusion and Exclusion Criteria

Criteria for inclusion of women in the study group: age over 18 (the age of majority in Poland), confirmation of endometriosis (ICD‐10 code, N80 with various extensions) and native language—Polish. The criteria for excluding respondents from the study: the age of women under 18 years, pregnancy, postmenopausal period, the presence of other chronic diseases, the course of which could affect the results of the study.

### 4.3 Study Setting and Sampling

After obtaining consent from the women to participate in the study, they were interviewed and their medical records were reviewed. The respondents then independently filled out a prepared survey questionnaire. To conduct the study, a diagnostic survey method was used based on a questionnaire technique. The tools were the Endometriosis Health Profile—EHP and a standardised interview questionnaire containing questions about the sociodemographic characteristics of women participating in the study. The questionnaire on respondents' characteristics included questions on continuous data: age (years) and categorical data—residence (urban, rural), relationship status (married, in a partnership, single), education (other than higher, higher), employment (employed, unemployed), socio‐economic standing (satisfying, not satisfying), having children—the question was for those trying for pregnancy (yes, not). Questions regarding medical data related to the diagnosis of the disease occurring in the last 6 months, included average duration of menstrual bleeding (days), the occurrence of: regular menstruation (yes, no), heavy menstruation (yes, no), painful menstruation (yes, no), pain during the cycle (yes, no), dyspareunia—the question was aimed at sexually active people (yes, no, I am not sexually active) and duration of treatment for endometriosis, hormonal treatment (yes, no), surgical treatment of endometriosis (yes, no).

### 4.4 Instrument

The Endometriosis Health Profile questionnaire (EHP‐30) developed by Jones et al. assesses the quality of life of women with endometriosis. It consists of two parts. The first includes a 30‐item baseline form, which applies to all women with endometriosis. The questions cover the last 4 weeks and the tool includes five scales on issues such as pain (11 items), control and helplessness (6 items), emotions (6 items), social support (4 items) and self‐assessment (3 items). The second is a modular form aimed at assessing the impact of the disease in the last 4 weeks in the following six areas: work life (5 items), relationship with children (2 items), cohabitation (5 items), feelings towards medical personnel (4 items) treatment (3 items) and infertility (4 items). The questionnaire is completed by respondents. Responses are rated on a 5‐point Likert scale (0—never, 1—rarely, 2—sometimes, 3—often and 4—always). The total score on each scale is from 0 to 100 points (each score is divided by the maximum number of points and then multiplied by 100). The smallest number of points indicates the best possible health status. Cronbach's α coefficient ranges from 0.83 to 0.93 (basic questionnaire) and 0.79 to 0.96 (modular questionnaire) (Jones et al. 2001).

### 4.5 Data Analysis

The collected material was statistically processed using the IBM SPSS Statistics package (v. 29). Quantitative variables were described by mean, standard deviation, median and also minimum and maximum values. For qualitative variables, the abundance and percentage of categories were given. Linear regression analysis was used to examine the effect of multiple independent variables (referred to as explanatory variables) on a single dependent variable (referred to as an explanatory variable). The independent medical variables were: average duration of bleeding, regular periods, heavy periods, painful periods, pain during the cycle, dyspareunia, treatment time for endometriosis and hormonal treatment. The dependent variables were, in turn EHP‐30 (total score) and scales: pain, control and powerlessness, emotional well‐being, social support, self‐image, work life, children, sexual intercourse, medical profession, treatment and infertility. The independent variables that are not quantitative were coded using the dummy coding method. According to it, a variable takes the value of 1 for some observations and 0 for others. We used the Shapiro–Wilk test to check the normal distribution. Skewness values were within ± 1 and kurtosis values were within ± 2. The assumptions of linearity and homogeneity of variance were checked using scatter plots and no heteroscedasticity/no clear pattern was found in the plots. Multicollinearity, the minimum and maximum variable inflation factor (VIF) were checked. A general *F*‐test and an adjusted *R*‐square were considered. Unstandardised beta regression coefficients (*B*) with 95% confidence intervals and standardised beta coefficients (*β*) were calculated to assess the level of association and statistical significance in the multiple regression analysis. The results of the analysis were considered statistically significant at *p* < 0.05.

### 4.6 Ethical Considerations

The study was approved by the Lublin Medical University Bioethics Committee (approval no. KE‐ 0254/256/2020). Respondents were informed that participation was voluntary, study results were anonymous and to be used exclusively for research purposes.

## 5 Results

### 5.1 Characteristics of the Sample

The mean (SD) age of the women surveyed was 31.07 (6.45) years, the majority of respondents lived in the city (69.4%), were married/in a stable relationship (58.1%), had a university degree (68.2%), worked professionally (72.7%), considered themselves to have satisfactory socioeconomic conditions (85.6%) and had no children (59.1%). Menstrual bleeding lasted an average of 6.20 (3.68) days. Most of the respondents declared that they had regular periods (64.2%), rated them as heavy (71.3%), the vast majority of women (92.9%) were accompanied by pain during menstruation, more than half of the women had pain during the cycle (54.35%) and pain during intercourse (58.3%). Most of the respondents had been treated for endometriosis for up to 2 years (56.0%), slightly more than half of the respondents had been treated with hormonal therapy (53.2%) and had undergone surgery for endometriosis (56.9%) (Table 1).

**TABLE 1:** Characteristics of the study group.

Characteristics of the group	*N*	%
Age		
M (SD) Me	31.07 (6.45) 30.00	
Residence		
Urban	295	69.4
Rural	130	30.6
Relationship status		
Married, in a partnership	247	58.1
Single	178	41.9
Education		
Other than higher	135	31.8
Higher	290	68.2
Employment		
Employed	309	72.7
Unemployed	116	27.3
Socio‐economic standing		
Satisfying	364	85.6
Not satisfying	61	14.4
Having children		
Yes	174	40.9
No	251	59.1
Average duration of bleeding during the cycle (days)		
M (SD) Me	6.20 (3.68) 6.00	
Regular periods		
Yes	273	64.2
No	152	35.8
Heavy periods		
Yes	303	71.3
No	122	28.7
Painful periods		
Yes	395	92.9
No	30	7.1
Discomfort during the cycle, such as pelvic pain, dysuria, dyschezia		
Yes	231	54.35
No	194	45.65
Dyspareunia		
Yes	248	58.3
No	147	34.6
I am not sexually active	30	7.1
Treatment time for endometriosis (years)		
Up to 2 years	238	56.0
3–4 years	64	15.1
5–10 years	76	17.9
Over 10 years	47	11.0
Hormonal treatment		
Yes	226	53.2
No	199	46.8
Surgical treatment of endometriosis		
Yes	242	56.9
No	183	43.1

Abbreviations: M, mean; Me, median; SD, standard deviation.

### 5.2 EHP‐30 Results

The mean (SD) rating 30.21 (26.65) of quality of life by women with endometriosis and individual components of quality of life by the women with endometriosis surveyed are presented in Table 2. Women's quality of life was rated lowest in the domains of infertility 55.46 (34.67), treatment 51.32 (30.51) and sexual intercourse 44.46 (32.28).

**TABLE 2:** Health assessment (EHP‐30) by women with endometriosis.

Variables	M	Me	SD	Min	Max	*A*	*K*
EHP‐30	30.21	26.74	26.65	0.00	82.16	0.615	−0.903
Pain	36.20	29.55	32.01	0.00	84.09	0.277	−1.475
Control and powerlessness	32.55	29.17	31.80	0.00	91.67	0.588	−1.010
Emotional well‐being	35.37	25.00	24.76	0.00	91.67	0.927	0.229
Social support	17.88	6.25	18.89	0.00	75.00	0.795	−0.387
Self‐image	29.04	25.00	33.06	0.00	91.67	0.636	−1.123
Work‐life	39.69	40.00	29.49	0.00	100.00	0.142	−1.153
Children	29.85	25.00	30.43	0.00	100.00	0.424	−1.245
Sexual intercourse	44.46	45.00	32.28	0.00	100.00	0.041	−1.268
Medical profession	43.61	43.75	32.85	0.00	100.00	0.004	−1.331
Treatment	51.32	50.00	30.51	0.00	100.00	−0.210	−1.034
Infertility	55.46	62.50	34.67	0.00	100.00	−0.407	−1.179

Abbreviations: *A*, skewness; *K*, kurtosis; M, mean; Me, median; SD, standard deviation.

### 5.3 Clinical Variables Related to the EHP‐30

Fewer heavy periods were associated with a higher quality of life (*β* = −0.141; *p* = 0.006), while painful periods (*β* = 0.111; *p* = 0.030), pain during intercourse (*β* = 0.192; *p* < 0.001) and surgical treatment of endometriosis (*β* = 0.111; *p* = 0.045) were associated with lower quality of life. Not having heavy periods was associated with a higher quality of life in the pain domain (*β* = −0.127; *p* = 0.013), while having painful periods (*β* = 0.117; *p* = 0.021), pain during intercourse (*β* = 0.183; *p* < 0.001) and having surgery for endometriosis (*β* = 0.150; *p* = 0.007) were associated with lower quality of life in this domain. The absence of heavy periods (*β* = −0.117; *p* = 0.022) was associated with a higher quality of life for the control and powerlessness domain, while having pain during intercourse (*β* = 0.194; *p* < 0.001) and having surgical treatment of endometriosis (*β* = 0.116; *p* = 0.036) were associated with lower quality of life. The absence of heavy periods (*β* = −0.145; *p* = 0.004) was associated with higher quality of life in the emotional well‐being domain, while the occurrence of painful periods (*β* = 0.120; *p* = 0.018) and pain during intercourse (*β* = 0.219; *p* < 0.001) were associated with lower quality of life in this domain. The absence of heavy menstruation (*β* = −0.109; *p* = 0.039) was associated with a better quality of life in the social support domain, but the presence of pain during intercourse (*β* = 0.148; *p* = 0.004) was associated with a lower quality of life in this domain. The absence of heavy periods (*β* = −0.161; *p* = 0.002) was associated with a higher self‐image domain quality of life and the occurrence of painful periods (*β* = 0.111; *p* = 0.031) and dyspareunia (*β* = 0.161; *p* = 0.002) were associated with a lower quality of life in this domain.

The occurrence of painful menstruation (*β* = 0.128; *p* = 0.014), pain during the cycle (*β* = 0.124; *p* = 0.026) and the occurrence of pain during intercourse (*β* = 0.231; *p* < 0.001) was associated with a lower quality of life on the work‐life domain. The presence of painful menstruation (*β* = 0.132; *p* = 0.049) and dyspareunia (*β* = 0.179; *p* = 0.007) were associated with worse quality of life in the children's domain. Dyspareunia was associated with a lower quality of life in the sexual intercourse domain (*β* = 0.669; *p* < 0.001), medical profession domain (*β* = 0.224; *p* < 0.001) and treatment domain (*β* = 0.229; *p* < 0.001) (Table 3).

**TABLE 3:** Regression analysis for subscales of the questionnaire for assessing the quality of life of women with endometriosis (EHP‐30) and medical variables.

Predictors	EHP‐30					Pain					Control and powerlessness				
	*F* = 3.794; *p* < 0.001 *R* = 0.328; *R* ^2^ = 0.108; adjusted *R* ^2^ = 0.079					*F* = 4.098; *p* < 0.001 *R* = 0.340; *R* ^2^ = 0.115; adjusted *R* ^2^ = 0.087					*F* = 3.844; *p* < 0.001 *R* = 0.330; *R* ^2^ = 0.109; adjusted *R* ^2^ = 0.081				
	*B*	*β*	*p*	LL	UL	*B*	*β*	*p*	LL	UL	*B*	*β*	*p*	LL	UL
(Constant)	20.038		0.006	5.826	34.251	23.519		0.007	6.479	40.559	21.222		0.014	4.300	38.144
The average duration of bleeding	−0.118	−0.017	0.734	−0.801	0.565	−0.259	−0.031	0.534	−1.078	0.559	−0.244	−0.029	0.555	−1.057	0.569
Regular periods	−3.750	−0.067	0.182	−9.260	1.761	−5.539	−0.082	0.100	−12.146	1.068	−5.727	−0.086	0.087	−12.289	0.834
Heavy periods	−8.286	−0.141	0.006	−14.209	−2.363	−9.025	−0.127	0.013	−16.127	−1.924	−8.243	−0.117	0.022	−15.295	−1.190
Painful periods	12.510	0.111	0.030	1.235	23.785	15.883	0.117	0.021	2.365	29.401	12.569	0.094	0.066	−0.855	25.994
Pain during the cycle	−1.406	−0.063	0.262	−3.864	1.052	−1.702	−0.063	0.257	−4.649	1.245	−1.027	−0.039	0.491	−3.954	1.899
Dyspareunia	1.531	0.192	< 0.001	5.084	15.979	12.119	0.183	< 0.001	5.587	18.650	12.687	0.194	< 0.001	6.201	19.174
Treatment time for endometriosis	0.038	0.030	0.548	−0.085	0.161	0.057	0.038	0.450	−0.091	0.204	0.038	0.026	0.607	−0.108	0.185
Hormonal treatment	3.424	0.064	0.200	−1.826	8.674	3.462	0.054	0.280	−2.833	9.757	4.492	0.071	0.158	−1.759	1.743
Surgical treatment of endometriosis	5.958	0.111	0.045	0.129	11.786	9.691	0.150	0.007	2.702	16.679	7.418	0.116	0.036	0.478	14.358

Predictors	Emotional well‐being					Social support					Self‐image				
	*F* = 4.202; *p* < 0.001 *R* = 0.343; *R* ^2^ = 0.118; adjusted *R* ^2^ = 0.090					*F* = 2.116; *p* = 0.015 *R* = 0.251; *R* ^2^ = 0.063; adjusted *R* ^2^ = 0.033					*F* = 3.138; *p* < 0.001 *R* = 0.301; *R* ^2^ = 0.091; adjusted *R* ^2^ = 0.062				
	*B*	*β*	*p*	LL	UL	*B*	*β*	*p*	LL	UL	*B*	*β*	*p*	LL	UL
(Constant)	24.348		< 0.001	11.301	37.395	11.989		0.023	1.650	22.328	19.114		0.035	1.390	36.839
The average duration of bleeding	0.069	0.011	0.828	−0.557	0.696	−0.033	−0.007	0.896	−0.530	0.464	−0.124	−0.014	0.775	−0.975	0.728
Regular periods	−1.642	−0.032	0.524	−6.701	3.417	−1.251	−0.032	0.540	−5.260	2.758	−4.589	−0.067	0.190	−11.461	2.284
Heavy periods	−7.912	−0.145	0.004	−13.349	−2.474	−4.546	−0.109	0.039	−8.855	−0.237	−11.707	−0.161	0.002	−19.093	−4.320
Painful periods	12.467	0.120	0.018	2.116	22.817	6.151	0.077	0.141	−2.051	14.353	15.481	0.111	0.031	1.420	29.542
Pain during the cycle	−1.760	−0.086	0.126	−4.017	0.496	−0.674	−0.043	0.459	−2.462	1.114	−1.865	−0.068	0.232	−4.931	1.200
Dyspareunia	11.128	0.219	< 0.001	6.127	16.129	5.788	0.148	0.004	1.825	9.751	1.934	0.161	0.002	4.140	17.728
Treatment time for endometriosis	0.016	0.014	0.776	−0.097	0.129	0.013	0.015	0.768	−0.076	0.103	0.063	0.041	0.417	−0.090	0.217
Hormonal treatment	2.472	0.050	0.314	−2.347	7.292	3.165	0.084	0.104	−0.654	6.985	3.529	0.054	0.290	−3.019	1.076
Surgical treatment of endometriosis	4.684	0.095	0.086	−0.668	1.035	1.721	0.045	0.425	−2.519	5.961	6.275	0.095	0.090	−0.994	13.544

Predictors	Work life					Children					Sexual intercourse				
	*F* = 4.725; *p* < 0.001; *R* = 0.370; *R* ^2^ = 0.137; adjusted *R* ^2^ = 0.108					*F* = 3.954; *p* < 0.001 *R* = 0.430; *R* ^2^ = 0.185; adjusted *R* ^2^ = 0.138					*F* = 24.667; *p* < 0.001 *R* = 0.675; *R* ^2^ = 0.455; adjusted *R* ^2^ = 0.437				
	*B*	*β*	*p*	LL	UL	*B*	*β*	*p*	LL	UL	*B*	*β*	*p*	LL	UL
(Constant)	11.834		0.147	−4.161	27.830	−12.858		0.225	−33.684	7.968	11.624		0.098	−2.139	25.387
The average duration of bleeding	0.375	0.049	0.326	−0.375	1.125	0.331	0.037	0.565	−0.800	1.462	0.202	0.024	0.547	−0.457	0.860
Regular periods	−4.850	−0.078	0.132	−11.165	1.465	−2.176	−0.034	0.597	−1.271	5.919	−1.659	−0.024	0.550	−7.112	3.794
Heavy periods	5.068	0.078	0.130	−1.500	11.636	−1.532	−0.022	0.738	−1.565	7.500	0.347	0.005	0.907	−5.506	6.200
Painful periods	15.579	0.128	0.014	3.221	27.936	15.454	0.132	0.049	0.100	3.807	1.600	0.012	0.776	−9.439	12.638
Pain during the cycle	2.144	0.085	0.128	−0.620	4.907	3.098	0.124	0.092	−0.509	6.705	1.166	0.043	0.347	−1.271	3.603
Dyspareunia	14.183	0.231	< 0.001	8.071	2.296	11.027	0.179	0.007	3.055	18.999	44.648	0.669	< 0.001	39.265	5.031
Treatment time for endometriosis	0.070	0.050	0.323	−0.069	0.208	0.000	0.000	0.997	−0.173	0.172	0.009	0.006	0.883	−0.117	0.135
Hormonal treatment	1.273	0.022	0.674	−4.674	7.219	4.864	0.080	0.225	−3.021	12.750	0.777	0.012	0.767	−4.379	5.934
Surgical treatment of endometriosis	−5.849	−0.098	0.076	−12.323	0.626	−3.116	−0.050	0.495	−12.110	5.879	0.909	0.014	0.755	−4.821	6.638

Predictors	Medical profession					Treatment					Infertility				
	*F* = 2.855; *p* < 0.001 *R* = 0.298; *R* ^2^ = 0.089; adjusted *R* ^2^ = 0.058					*F* = 2.757; *p* = 0.001 *R* = 0.307; *R* ^2^ = 0.095; adjusted *R* ^2^ = 0.060					*F* = 8.226; *p* < 0.001 *R* = 0.496; *R* ^2^ = 0.246; adjusted *R* ^2^ = 0.216				
	*B*	*β*	*p*	LL	UL	*B*	*β*	*p*	LL	UL	*B*	*β*	*p*	LL	UL
(Constant)	23.327		0.011	5.369	41.286	23.958		0.008	6.161	41.756	48.785		< 0.001	29.500	68.070
The average duration of bleeding	0.358	0.043	0.408	−0.492	1.208	0.204	0.027	0.615	−0.592	1.000	0.324	0.039	0.446	−0.512	1.160
Regular periods	−4.438	−0.065	0.214	−11.444	2.567	−3.259	−0.052	0.352	−1.136	3.617	−2.650	−0.036	0.481	−1.041	4.740
Heavy periods	−2.743	−0.038	0.481	−1.393	4.908	2.382	0.036	0.526	−5.002	9.766	1.113	0.015	0.777	−6.625	8.852
Painful periods	11.721	0.086	0.110	−2.668	26.111	9.572	0.076	0.182	−4.515	23.658	9.469	0.064	0.218	−5.636	24.574
Pain during the cycle	1.806	0.066	0.264	−1.372	4.984	1.562	0.059	0.335	−1.623	4.747	2.982	0.101	0.080	−0.364	6.328
Dyspareunia	14.974	0.224	< 0.001	7.999	21.949	14.324	0.229	< 0.001	7.525	21.123	5.843	0.082	0.111	−1.349	13.035
Treatment time for endometriosis	0.062	0.040	0.448	−0.099	0.224	−0.080	−0.046	0.412	−0.270	0.111	0.043	0.027	0.598	−0.117	0.202
Hormonal treatment	0.691	0.011	0.840	−6.028	7.410	3.989	0.066	0.232	−2.562	1.540	−2.831	−0.041	0.424	−9.791	4.129
Surgical treatment of endometriosis	−1.851	−0.028	0.628	−9.351	5.649	2.928	0.048	0.422	−4.230	1.086	−5.303	−0.076	0.177	−13.012	2.406

Abbreviations: *β*, standardised coefficient; *B*, unstandardised coefficient; LL, lower limit (95% CI); UL, upper limit (95% CI).

## 6 Discussion

Endometriosis is a complex condition that significantly affects the lives of women who suffer from it. The consequences of endometriosis affect the entire life cycle, from the onset of symptoms through the decades of a woman's life. They can hinder education, limit job performance, alter career choices and success, impair social life and activities, affect plans and family choices, create tension in personal relationships, negatively affect mental and emotional health (Rush and Misajon 2018; Missmer et al. 2021). Managing the symptoms of endometriosis requires a multidisciplinary approach that can include pain management strategies, hormonal therapies, and in severe cases, surgical intervention. Despite such management, negative effects on women's quality of life can persist, underscoring both the need for ongoing research and improved therapeutic options. The psychological effects and difficulties of long‐term medical care or infertility, for example, can be severe in addition to the physical symptoms (Gallagher et al. 2018; Rodrigues et al. 2022). In our study, the general quality of life assessment of endometriosis‐affected women was higher than in studies conducted among women living in the United States, Swedish women and at a similar level in Dutch women (Soliman et al. 2017; Pontoppidan, Olovsson, and Grundström 2023; Apers et al. 2018). This result could be caused by the fact that the time of treatment of the disease in just over half of the respondents was up to 2 years. The relatively short treatment time for endometriosis may result in women having more hope for a positive course of treatment and relief of symptoms, which may affect their well‐being and quality of life. Analysis of individual domains of quality of life showed that the respondents rated quality of life lowest in the dimensions of infertility (even though 40.9% of respondents had children), need for treatment and sexual intercourse.

### 6.1 Infertility

One of the biggest challenges facing women with endometriosis is the increased risk of infertility. The psychological consequences of infertility might exacerbate endometriosis patients' already low quality of life. The mean (SD) age of the respondents was 31.07 (6.45). This is a time of life when many women are planning motherhood or actively trying to have a child. At this age, the desire to get pregnant can be even more acute, especially for women with endometriosis, who have an increased risk of infertility. Women who want to get pregnant confront mental and physical challenges that require specific treatment and interventions. Endometriosis‐related infertility is a huge source of frustration for women and their partners. The longer the condition lasts, the worse it becomes (Heng and Shorey 2022; Missmer et al. 2021; Mori et al. 2024). Due to biological factors, the longer the infertility persists, the more difficult it can be for women to accept their inability to conceive (Hudson et al. 2016). Furthermore, when assisted reproduction techniques are required, a patient's age can influence the effectiveness of treatment, which can further intensify fertility concerns in this age group (Mori et al. 2024). Especially, as Macer et al. point out, the population of infertile women with endometriosis is heterogeneous and a variety of patient phenotypes can be observed in the clinical setting, making it difficult to establish an accurate diagnosis and unambiguous mechanism of endometriosis‐related infertility. Moreover, clinical management of endometriosis‐related infertility can be challenging due to this heterogeneity (Macer and Taylor 2012).

### 6.2 Therapeutic Process

Women with endometriosis need to undergo a demanding and diverse therapy process for the rest of their lives to keep the condition under control (Jones et al. 2024). Endometriosis treatment frequently needs a multimodal approach, which may include lifestyle changes, medication and surgery. The main goal of treatment is pain management, for which non‐steroidal anti‐inflammatory drugs (NSAIDs) and hormonal therapies are commonly recommended. In cases where negative symptoms are exacerbated or there is a concern about fertility, surgical intervention may be necessary to remove endometrial and scar tissue. Treatment for endometriosis‐related infertility can include the use of assisted reproductive technology (ART). The need for chronic use of medications in therapy can reduce women's quality of life. It also raises some issues, such as the risk of unintentionally failing to follow the prescribed drug dosage regimen, malaise due to possible side effects, costs incurred for medications and concerns about the lack of effectiveness of treatment (Vermeulen et al. 2021; Mori et al. 2024). In addition to medical interventions, the need to modify lifestyles including diet, physical activity, physiotherapy or therapies aimed at coping with stress, on the one hand, is aimed at improving women's health, but on the other hand, it somehow forces women to live in a specific way (Mazur‐Bialy et al. 2024; Lalla et al. 2024; Oszajca and Adamus 2024). These actions can have positive and negative connotations. From one perspective, making changes that can alleviate the symptoms of the disease can be seen as a necessity, a challenge and an opportunity to improve overall health. In contrast, these changes may create psychological strain, additional stress or even the need to change life priorities (relationships, career) associated with the necessary activities that women must take to improve their health. These activities are also related to the financial burden of incurring additional costs for therapies. In addition to the costs directly related to the treatment of endometriosis, women may incur additional expenses related to frequent medical visits, diagnostic examinations, for example, USG, MRI, the purchase of painkillers, hygiene products, pelvic floor therapy, infertility treatment with IVF procedure and other costs associated with maintaining health (Abril‐Coello et al. 2023).

### 6.3 Surgical Treatment

Studies on therapies for endometriosis and their impact on quality of life indicate that surgery significantly improves women's health‐related quality of life, compared to medical treatment. (Jones et al. 2024; Nogueira Neto et al. 2023; Poordast et al. 2022; Tiringer et al. 2022). Differences are particularly noticeable in the first weeks after surgery, although according to Vercellini et al. 1 year after surgery, quality of life improves in both groups (treated conservatively and surgically), despite the method of treatment (Vercellini et al. 2013). In contrast, the results of our study showed that surgery for endometriosis influences lower general EHP‐ 30 scores, as well as worse scores in the pain and control and powerlessness categories. Whereas surgery may be required to address disease‐related discomfort, the process can induce pain and discomfort, significantly impacting quality of life. Surgery can result in a loss of control over one's body and health situation, which can contribute to a lower quality of life in terms of controlling one's destiny. Furthermore, undergoing surgery for endometriosis can result in additional psychological and emotional stress, which can have a severe impact on coping with pain and patients' sense of control (Samami et al. 2023). Women may also be aware that endometriosis is a chronic disease, and even after surgery, regression of symptoms is not always certain. This can lead to feelings of helplessness and lack of control over health (Bougie et al. 2021; Long et al. 2023).

### 6.4 Pain During the Cycle

According to our findings, the most common problem reported by women with endometriosis is painful and heavy menstruation. These variables determine poorer general quality of life in women and lower quality of life in the domains of pain, emotional well‐being and self‐image. Endometriosis is the most common cause of secondary painful menstruation in adult and adolescent women and clinical manifestations may differ among age groups, which may result in delayed diagnosis of the disease. Painful menstruation is a leading cause of short‐term absenteeism from school in adolescent girls. Observation of the nature of bleeding, its severity and pelvic pain during menstruation from the onset of the first menstrual period, makes it possible to identify several symptoms that may herald the presence of endometriosis and other fertility problems (Gutman, Nunez, and Fisher 2022; Sachedina and Todd 2020). The presence of painful and heavy menstruation, in addition to poorer physical well‐being, also determines women's poorer emotional well‐being. As Van Niekerk et al. point out, a defence mechanism for mental health in women with endometriosis may be to adopt a forgiving attitude towards the disease's symptoms. Especially when women with more pain‐related endometriosis symptoms are more likely to evaluate themselves negatively which reduces self‐compassion. (Van Niekerk, Johnstone, and Matthewson 2022).

Proper pain and disease‐related symptoms management can reduce the consequences associated with anxiety and depression at the same time also improving women's quality of life. Due to the interaction between somatic and psychological symptoms of endometriosis, it may be beneficial to use relaxation techniques or cognitive behavioural therapy (CBT) to alleviate depression, stress and reduce pain sensation (Mazur‐Bialy et al. 2024). Pharmacological and surgical treatment can also help relieve pain, particularly discomfort associated with menstruation, and thus improve the physical, mental and social quality of life in women even in the long term (Yela, Quagliato, and Benetti‐Pinto 2020; da Cunha Araújo et al. 2014; Vannuccini et al. 2019).

Painful menstruation can also lead to frustration and loss of control over one's own body, which can significantly influence self‐esteem, generating thoughts of unreliability or imperfection (Berger et al. 2019; Nassiri Kigloo et al. 2024). This is a potential explanation for the obtained correlations between painful menstruation and low quality of life in the self‐image domain, which relates to perceptions of one's attractiveness and self‐confidence. The results of our study also confirmed that pain caused by the disease also affects poorer quality of life in the context of work life. Patients diagnosed with endometriosis have to cut their work hours, change jobs or even resign from school or work because of lower productivity due to the chronic nature of the pain and other symptoms, such as fatigue, heavy bleeding and mood swings (Nnoaham et al. 2011; Andysz et al. 2018). Loss of productivity or frequent absenteeism from work among women affected by the condition is linked to both of the aforementioned occupational problems, as well as the necessity to stop work, which can result in loss of income and additional financial difficulties (De Graaff et al. 2013). In contrast, Gremillet et al. indicated that, according to women, cyclical pelvic pain is a discomfort when it occurs during professional activity; at the same time, for more than half of the respondents it is an embarrassing disease (especially if endometriosis was accompanied by infertility treatment), so they kept quiet about it in the workplace because of shame, fear of being judged by others (Gremillet et al. 2023).

### 6.5 Dyspareunia

Dyspareunia affects the quality of life of women with endometriosis, causing sex to be an extremely painful experience for women (Jimenez et al. 2023; Wahl et al. 2021). A common cause of it is endometriosis foci located in the pouch of Douglas, which should be removed surgically to relieve symptoms. Painful sexual intercourse can result in decreased sexual satisfaction, which in turn can lead to sexual aversion. However, in a situation where women are trying to get pregnant, they can pretend that sexual intercourse is painless, as the desire to have a child comes first (Melis et al. 2015). This, in turn, contributes to the interruption of intercourse, reluctance to engage in sexual contact or the search for alternative ways to please oneself and one's partner (Jimenez et al. 2023; Melis et al. 2015). It may affect women's mental health, self‐esteem and sexual quality (Missmer et al. 2021; Jimenez et al. 2023; Fritzer et al. 2014; Wahl et al. 2021; Merli et al. 2024; Florentino et al. 2019). The results of our study are consistent with those presented above, as experiencing dyspareunia among individuals with endometriosis was associated with lower overall EHP‐30 scores, as well as in many domains (pain, control and helplessness, emotional well‐being, social support, body image, work life, children, sexual relations, medical profession and treatment). Therefore, in the current perspective, early detection and further treatment of dyspareunia appear to be essential to ensure that women with endometriosis receive adequate treatment from a multidisciplinary team of health care representatives (Jimenez et al. 2023; Merli et al. 2024).

### 6.6 Strengths and Limitations of the Work

This study used a unique tool to assess general quality of life and evaluate individual domains related to quality of life in women with endometriosis. Thus, this tool can compare the results obtained with other authors. The strength of our study is that we used the second part of the EHP‐30 questionnaire, which researchers less frequently use, the so‐called modular form assessing the impact of the disease on additional areas of women's lives: work life, relationships with children, cohabitation, feelings towards medical personnel, treatment and infertility. This provided a more complete picture of the disease's effects on women with endometriosis. Assessing extra areas of life allows us to better understand patients' individual needs and personalise therapy to their ailments. In addition, focusing on areas of life that are less frequently examined can reveal additional challenges faced by women affected by the disease.

As with all studies, this study has some limitations. In our study, we did not collect data on the clinical stage or type of endometriosis. Secondly, the heterogeneity of respondents' age may affect the results obtained. Finally, the cross‐sectional approach captures symptoms and burden at a single point in time and does not provide an accurate picture of longitudinal burden. Given, the unpredictable nature of disease exacerbations, it would make sense to examine the burden of endometriosis over time. Nevertheless, we are confident that this study contributes to the knowledge and understanding of the burdening effects of the disease on women's quality of life.

### 6.7 Recommendations for Further Research

A prospect for further research is to identify a cutoff point for EHP‐30 scores to realistically determine the level of quality of life in women with endometriosis. This would be a useful tool for determining the value of the surgical or hormonal treatment used and its impact on patients in specific domains. The EHP‐30 tool would then have greater predictive value in developing a management strategy for each case. Pontoppidan et al. emphasise the importance of patient‐centred care, showing that a higher level of patient focus was associated with better quality of life in a group of women with endometriosis (Pontoppidan, Olovsson, and Grundström 2023).

### 6.8 Implications for Policy and Practice

Endometriosis significantly affects the quality of life of women affected by it, especially when infertility, the need for treatment or emerging problems in sexual life become the main concerns. These findings highlight the need for more targeted interventions in these areas to improve women's quality of life. These include providing better access to treatment, including refunding hormone therapies, pain killers and alternative therapies that can help alleviate the symptoms of the disease; giving women quicker access to free specialised gynaecological care, including fertility care to increase their chances of getting pregnant. Increasing the availability of therapy sessions and support groups for women with endometriosis can help them cope with the emotional and psychological effects of the disease; creating educational programs that provide information about endometriosis, its symptoms, available therapies and coping strategies. Due to the scale of the disease and the difficulties women face in functioning in their daily lives, as well as at work, national information campaigns are recommended to raise awareness of endometriosis, its symptoms and its impact on women's lives. It seems intentional to introduce a policy that makes it easier to obtain sick leave for women who have severe menstrual pain or heavy bleeding. Shorter work days or the possibility of remote work should be considered. Implementing these recommendations, supporting research initiatives on the disease and promoting open conversations about endometriosis can contribute to a better quality of life.

Endometriosis is a condition with etiological factors that are not yet fully understood, which complicates the development of definitive preventive strategies. However, certain measures may potentially reduce the risk of developing this disease or alleviate its symptoms. One such measure is the promotion of a healthy lifestyle, which includes regular physical activity and a balanced diet. Educating women, including adolescents, about the symptoms of endometriosis and encouraging regular gynaecological check‐ups may aid in early detection. Access to available screening tests can facilitate prompt diagnosis and intervention. It is important to note that while these strategies may assist in the prevention or early detection of endometriosis, they do not guarantee complete protection against the condition. Therefore, regular health monitoring and consultations with a gynaecologist are crucial in the presence of concerning symptoms.

## Author Contributions

A.B., A.P., K.J. made substantial contributions to conception and design or acquisition of data or analysis and interpretation of data; A.B., A.P., J.G.‐G., M.K.‐P., M.Z., E.R. were involved in drafting the manuscript or revising it critically for important intellectual content; A.B., A.P., J.G.‐G., M.K.‐P., M.Z., E.R., K.J. gave final approval of the version to be published. Each author should have participated sufficiently in the work to take public responsibility for appropriate portions of the content; A.B., A.P., J.G.‐G., M.K.‐P., M.Z., E.R., K.J. agreed to be accountable for all aspects of the work in ensuring that questions related to the accuracy or integrity of any part of the work are appropriately investigated and resolved.

## Conflicts of Interest

The authors declare no conflicts of interest.

## Peer Review

The peer review history for this article is available at https://www.webofscience.com/api/gateway/wos/peer‐review/10.1111/jan.16607.

## Data Availability

The data that support the findings of this study are available from the corresponding author upon reasonable request.
